# Multiple Cream-Colored Papules Over the Trunk and Neck

**DOI:** 10.4103/0974-2077.58528

**Published:** 2009

**Authors:** S Vijay Shankar, KC Nischal, MR Swaroop, HB Basavaraj, BD Sathyanarayana

**Affiliations:** *Department of Pathology, Adichunchanagiri Institute of Medical Sciences, BG Nagar, Karnataka, India*; 1 *Department of Dermatology, Adichunchanagiri Institute of Medical Sciences, BG Nagar, Karnataka, India*

A 55 year-old male presented with asymptomatic, tiny, raised lesions of six years' duration on the chest, abdomen, and back. These lesions had gradually increased in size and number. None of the family members were affected.

Examination revealed cream-colored, dome-shaped papules of 2-5 mm size over the root of the neck, chest [Figure 1], abdomen, back [Figure 2], and also on the medial arms. The lesions did not have any punctum. A small skin-colored papule of 2 mm size was present on the right scrotal wall. Nails were normal.

**Figure 1:**
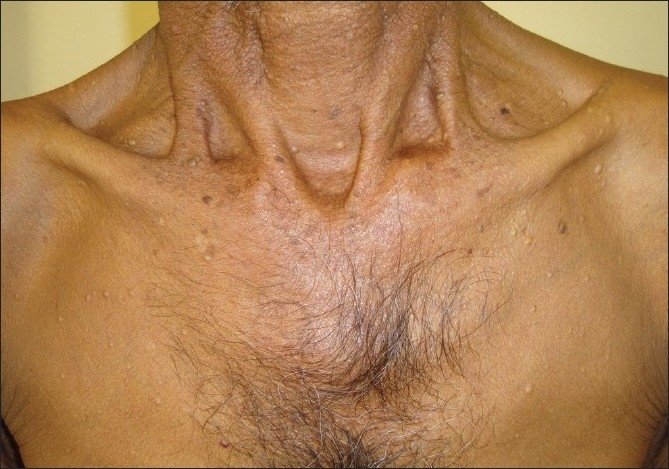
Multiple cream-colored papules on the chest and neck

**Figure 2:**
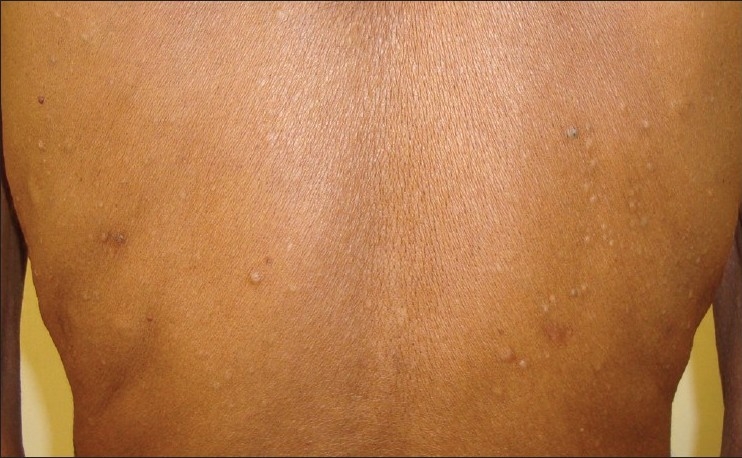
There are few comedones in addition to the creamish papules on the back

An excisional biopsy was performed from a papule from the back; the histological picture is seen in Figures 3 and 4.

**Figure 3:**
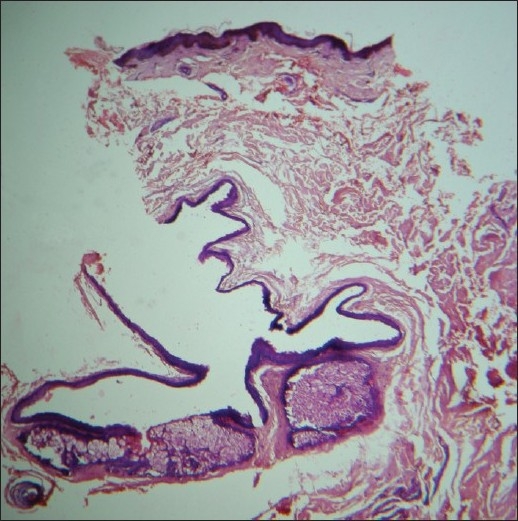
Collapsed cyst whose walls are thrown into folds and lined by squamous epithelium (H and E, ×40)

**Figure 4:**
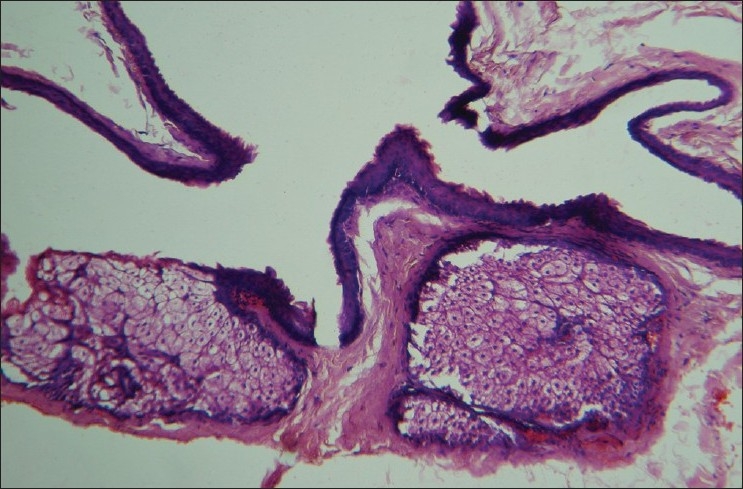
Higher magnification of the cyst wall (H and E, ×400)

## WHAT IS YOUR DIAGNOSIS?

## DIAGNOSIS: STEATOCYSTOMA MULTIPLEX

Histology revealed a collapsed cyst in the dermis. The cyst wall was lined by multiple layers of stratified squamous epithelium [Figure 3]. Lobules of sebaceous glands could be seen in the wall of the cyst. The luminal surface of the cyst wall revealed homogeneous eosinophilic cuticle at foci [Figure 4].

## DISCUSSION

Steatocystoma multiplex (sebocystomatosis) is an autosomal dominant condition[1] that usually presents in adolescence. It is characterized by multiple, yellowish or skin-colored, dome-shaped nodules and cysts over the trunk, proximal extremities, and the face. However, acral distribution has also been reported.[2] The common sites of involvement are the chest and the arms, but these nodules can occur anywhere in the body. Sometimes, the condition can manifest as a solitary lesion, when it is called steatocystoma simplex.

These papules have to be clinically differentiated from other cysts. Eruptive vellus hair cysts are commonly found on the chest. Vellus hair cysts are reddish brown in color and may be associated with itching. Epidermoid cysts and occasionally, vellus hair cysts may have a black punctum. Trichilemmal cysts are more common on the scalp.[3,4]

As the clinical features are variable, diagnosis may be difficult and require biopsy to be confirmed.

**Figure 5:**
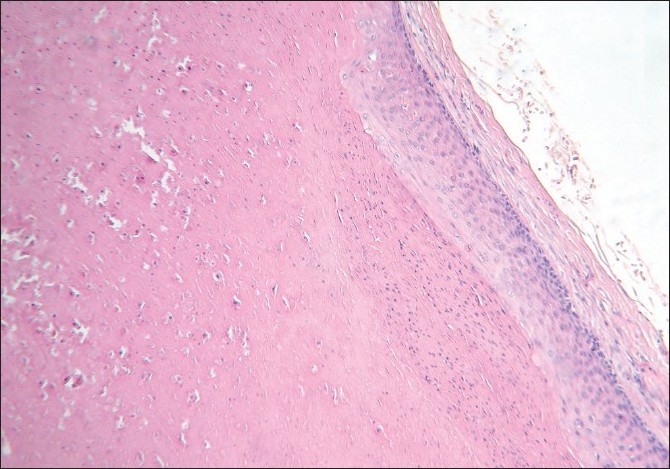
Trichilemmal cyst contains homogenous, eosinophilic, compact, orthokeratotic material within the sac and the lining epithelium characteristically lacks granular layer

**Figure 6:**
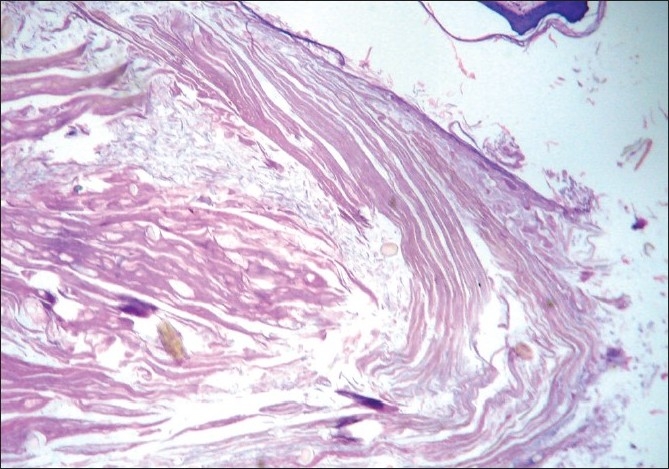
Typical vellus hair cyst: Admixed with the lamellated keratin, there are numerous vellus hairs, which is very typical of vellus hair cyst

**Figure 7:**
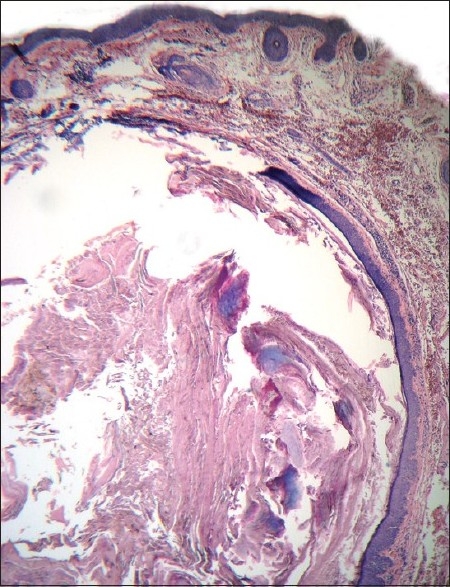
Infundibular cyst: Cyst wall is composed of stratified squamous epithelium with granular layer. Note the lamellated keratin in the cavity

Steatocystoma multiplex and simplex arise from the ducts of sebaceous glands.[5] Histologically, these cysts are characterized by folded and crenated cyst walls that are lined by several layers of stratified squamous epithelium with wavy eosinophilic cuticles towards the luminal surface.[6] In atrophic areas, the lining may be one to three layers of flattened squamous epithelium. The diagnostic feature is the presence of lobules of sebaceous glands, which are either close to or seen within the lining epithelium. Occasionally, the lining of the cyst wall may contain macrophages.[1] Sabater-Marco *et al*. have reported the presence of smooth muscles in the cyst wall. [7] These cysts are usually empty as the predominantly lipid contents are washed away during the processing of the biopsy specimen. Uncommonly, homogeneous, light-staining, eosinophilic contents and vellus hairs may be visualized in the cavity of the cysts. Steatocystoma has to be differentiated histologically from trichilemmal cysts [Figure 5], eruptive vellus hair cysts [Figure 6], and infundibular cysts [Figure 7].

Table 1 gives the salient features of different types of cutaneous cysts.

**Table 1:** Clinical and histopathological features of various follicular cysts

Cysts	Epidermoid/Sebaceous cyst	Trichilemmal/Pilar cyst	Steatocystoma	Eruptive vellus hair cyst
Age	Young and middle-aged	Young and middle-aged	Adolescence	Children and young adults
Distribution	Face, neck and upper trunk	Scalp	Trunk, upper extremities	Parasternal area
Morphology	Smooth dome-shaped swelling	Smooth dome-shaped swelling	Small dome-shaped translucent or yellowish	Soft, flesh-colored or reddish brown papules
Punctum	Present	Absent	Absent	May be present
Origin	Infundibulum of hair follicle	Isthmus of hair follicle	Duct of sebaceous glands	Arise in a developmental defect of hair follicle at the level of the infundibulum
Granular layer in the lining epithelium	Present	Absent	Absent	Present
Contents	Lamellated keratin	Homogeneous keratin	Most of the times, it is empty. If present, it is homogeneous and lightly stained.	Lamellated keratin with fine vellus hairs
Relation to the sebaceous glands	None	None	Lobules of sebaceous glands are seen within the lining epithelium or in close approximation to the cyst wall	None
Immunohistochemistry	Express K10	Express K10 and K17	Express K10 and K17	Express K17

Steatocystoma is considered as forme fruste of pachyonychia congenita II and has been reported to be associated with mutations of *keratin 17* gene in familial disease cases.[8‐10] Steatocystoma has been found in association with various conditions like multiple keratoacanthomas, rheumatoid arthritis, hyperkeratotic lichen planus, hidradenitis suppurativa, acrokeratosis verruciformis of Hopf, ichthyosis, koilonychias, and bilateral preauricular sinuses.[11,12]

Various modalities of treatment such as surgery, laser, and cryotherapy have been employed in treating this condition. Most of the surgical techniques involve excision of the cyst *in toto* or decapitation of the cyst, extirpation of its contents, and removal of the cyst wall. Cysts are opened by perforating the cysts with a needle tip, or by stab incision with a surgical blade no. 11, or by electrocautery, or by CO_2_ laser. [13‐18] The contents are then expressed out and the cyst wall removed or destroyed competely to prevent recurrence. Cyst walls can also be destroyed chemically or by CO_2_ laser or electrosurgery.[16] Isotretinoin (1 mg/kg/day) has been used with variable results in extensive steatocystomas.[19,20]
